# Therapeutic Pearls at Random Strung

**Published:** 1881-05

**Authors:** 


### THERAPEUTIC PEARLS AT RANDOM STRUNG.

### Whooping Cough.

| | | |
|---|---|---|
| ℞ | Bromide Potash, . . . . . . | ʒ i |
| | Biomide Ammonicum, . . . . . | ℥ iij |
| | Iodide Potash, . . . . . . . | ʒ ij |
| | Bicarbonate Patash, . . . . . . | ʒ i |
| | Fld. Ext. Cimicifuga, . . . . | |
| | Glycerine, . . . . . . . | aa ℥ iij Mix. |

Dose, a teaspoonful three times a day, and a dessertspoonful at bed time.

ANOTHER.

| | | |
|---|---|---|
| ℞ | Ioduret of Silver, . . . . . | grs. xi. |
| | White Sugar, . . . . . . | grs. lxx. |
| | Pulv. Gum Tragacanth, . . . . . | grs. x. |

Rub well together, and divide into eighty pills. S. To a child, *one* to *three* years old, one pill three times a day.

DR. MOTT'S PRESCRIPTION IS:

| | | |
|---|---|---|
| ℞ | Med; Hydrocyanic Acid, . . . . | gtt. vj. |
| | Ext. Belladonna, . . . . . | grs. ij. |
| | Paregoric and Water, each, . . . . | ℥ iij. |
| | Symp. Tolu, . . . . . . . | f. ℥ j. |
| | | M. S. |

A teaspoonful three times a day. It is said that a few grs. of Bromide of Potash enhances the efficacy of the prescription.

HEMORRHAGE, (Pancosts Styptic.)

| | | |
|---|---|---|
| ℞ | Potassæ Carbonatis, . . . . . . | ʒ i |
| | Saponis Castile, . . . . . . | ʒ ij |
| | Alcohol, . . . . . . . . . | ℥ iv M. |

Said to be preferable to Monsuls solution in arresting hemorrhage.

WHOOPING COUGH.

| | | |
|---|---|---|
| ℞ | Potassæ Bicarb, . . . . . | grs. xij |
| | Coce, Cacti, . . . . . . . | grs. x |
| | Potass. Bromide, . . . . . | grs. xvj |
| | Tinct Belladonæ, . . . . . | gtt. xx |
| | " Cardamon Camp. . . . . . | f. ℥ j |
| | Aquæ Cinnami, q. s. . . . . . | f. ℥ ij M. |

S. One teaspoonful every 3 or 4 hours for a child one or two years old, said to be very effective--almost specific.

HEADACHE, (Sick)

| | | |
|---|---|---|
| ℞ | Granulated Muriate of Amonia, one teaspoonful. | |
| | Acetate of Morphia, . . . . . | gr. j |
| | Water, . . . . . . . . . | f. ℥ viij M. |

S. Two teaspoonfuls, for an adult every 10 minutes (precisely) till relief is obtained.

COLD IN THE HEAD.

| | | |
|---|---|---|
| ℞ Hydrochlorate Morphia, . . . | grs. ij | |
| Pulv. Asacia, . . . . . . . | ʒ ij | |
| Trisnitrate Bismuth, . . . . | ʒ vj | M. |

Used as *snuff* several times a day, is highly recommended by Dr. Ferrier, of King's College, London.

